# A Novel β-Hairpin Peptide Z-d14CFR Enhances Multidrug-Resistant Bacterial Clearance in a Murine Model of Mastitis

**DOI:** 10.3390/ijms23094617

**Published:** 2022-04-21

**Authors:** Xue Wang, Shuxian Li, Mengze Du, Ning Liu, Qiang Shan, Yunjing Zou, Jiufeng Wang, Yaohong Zhu

**Affiliations:** 1 College of Veterinary Medicine, China Agricultural University, Beijing 100193, China; ivywang0913@163.com (X.W.); li_shuxian2021@163.com (S.L.); nliu2224@163.com (N.L.); xiaoqiangdebaobao@163.com (Q.S.); yunjingzouz@163.com (Y.Z.); jiufeng_wang@hotmail.com (J.W.); 2 Animal Science and Technology College, Beijing University of Agriculture, Beijing 102206, China; dumengze@126.com

**Keywords:** antimicrobial peptide, β-hairpin, defensin, *Zophobas atratus*, multidrug-resistant bacteria, mastitis

## Abstract

The widespread prevalence of antimicrobial resistance has spawned the development of novel antimicrobial agents. Antimicrobial peptides (AMPs) have gained comprehensive attention as one of the major alternatives to antibiotics. However, low antibacterial activity and high-cost production have limited the applications of natural AMPs. In this study, we successfully expressed recombinant *Zophobas atratus* (*Z. atratus*) defensin for the first time. In order to increase the antimicrobial activity of peptide, we designed 5 analogues derived from *Z. atratus* defensin, Z-d13, Z-d14C, Z-d14CF, Z-d14CR and Z-d14CFR. Our results showed that Z-d14CFR (RGCRCNSKSFCVCR-NH_2_) exhibited a broad-spectrum antimicrobial activity to both Gram-positive bacteria and Gram-negative bacteria, including multidrug-resistant bacteria. It possessed less than 5% hemolysis and 10% cytotoxicity, even at a high concentration of 1 mg/mL. Antimicrobial mechanism studies indicated that Z-d14CFR performed antimicrobial effect via inhibiting biofilm formation, disrupting bacterial membrane integrity and inducing cellular contents release. Furthermore, Z-d14CFR showed a great therapeutic effect on the treatment of multidrug-resistant *Escherichia coli* (*E. coli*) infection by enhancing bacterial clearance, decreasing neutrophils infiltration and the expression of tumor necrosis factor-alpha (TNF-α) and interleukin-1 beta (IL-1β) in a murine model of mastitis. Our findings suggest that Z-d14CFR could be a promising candidate against multidrug-resistant bacteria.

## 1. Introduction

In the last two decades, antimicrobial resistance has become a great threat to public health of the world due to the abuse of antibiotics [1,2]. It was reported that there were 700,000 people dead annually due to multidrug-resistant (MDR) bacterial infections, resulting in approximately $100 trillion economic losses [3]. However, antibiotics are currently the primary drugs for bacterial infection treatment, and the rise of MDR bacteria has still developed alarmingly [4]. According to the World Health Organization (WHO), the growing levels of antibiotic resistance will leave patients in a situation with no cure for medicine, therefore, it is necessary to develop new drugs to inhibit the rapid evolution of multidrug resistance [5,6].

In recent years, antimicrobial peptides (AMPs) have emerged as promising therapeutics against MDR pathogens due to their broad-spectrum antimicrobial activities, low drug resistance and toxicity, unique mechanism, and biological diversity [7,8]. Different from traditional antibiotics, AMPs mainly interact non-specifically with microbial membranes, and directly attack pathogens, therefore resulting in rapid and physical death of pathogens. It makes AMPs highly sensitive to MDR bacteria and largely avoids the development of antibacterial resistance [9,10]. To date, more than 3000 AMPs have been stored in the antimicrobial peptide database (APD, http://aps.unmc.edu/AP/) (Accessed: 20 March 2022). Unfortunately, only a few AMPs have entered clinical trial, such as pexiganan (MSI-78) and colistin [11,12,13].

The natural AMPs are generally featured with complicated extraction process, low antimicrobial activity and stability, high production costs and side-effects that extremely limit their clinical applications. Designing and synthesizing AMPs by genetic engineering technology can rationally modify the physicochemical property of AMPs in favor of their clinical application [6,14].

Insect defensins are ancient natural AMPs with strong antimicrobial activity against a wide range of microorganisms. They are generally Arg-rich cationic peptides with an amphiphilic α-helix and β-sheet, which are stabilized by three disulfide bonds [15,16]. The amphiphilic α-helix or β-sheet of insect defensin can constitute an independent motif with antimicrobial activity [17,18,19,20,21]. Peptides derived from insect defensins α-helix (such as sapecin B, coprisin, protaetiamycin, adefensin and ordefensin) and β-sheet (such as tenecin-1, lucifensin and navidefensin2-2) have been confirmed to exhibit antimicrobial activity, thus, these natural active regions were often used to design antimicrobial agents.

Our previous study suggested that natural *Z. atratus* hemolymph, which exhibited broad-spectrum antimicrobial activity and favorable safety in vitro, was a promising candidate for antibiotics alternative. Mass spectrometry analysis found that *Z. atratus* hemolymph contains multiple antibacterial substances, such as defensin [22,23]. However, the bioavailability of natural *Z. atratus* hemolymph is limited, and no systematic study about *Z. atratus* defensin has been reported. In this study, in order to overcome the low yield and slight antibacterial activity of natural *Z. atratus* hemolymph, we expressed recombinant *Z. atratus* defensin, and then designed five peptides derived from *Z. atratus* defensin β-sheet, based on its structural similarity to protegrin-1 (PG-1), which is a favorable β-hairpin antimicrobial peptide isolated from porcine leukocytes [24]. Among the synthesized peptides, Z-d14CFR exhibited increased antibacterial activity against both Gram-positive and Gram-negative bacteria, including multidrug-resistant bacteria. Subsequently, we preliminarily investigated the antimicrobial mechanism of Z-d14CFR and explored its possibility for the treatment of MDR bacterial infection. We attempted to provide implications for novel antimicrobial drug development.

## 2. Results

### 2.1. Recombinant Z. atratus Defensin Expression

**Figure 1:**
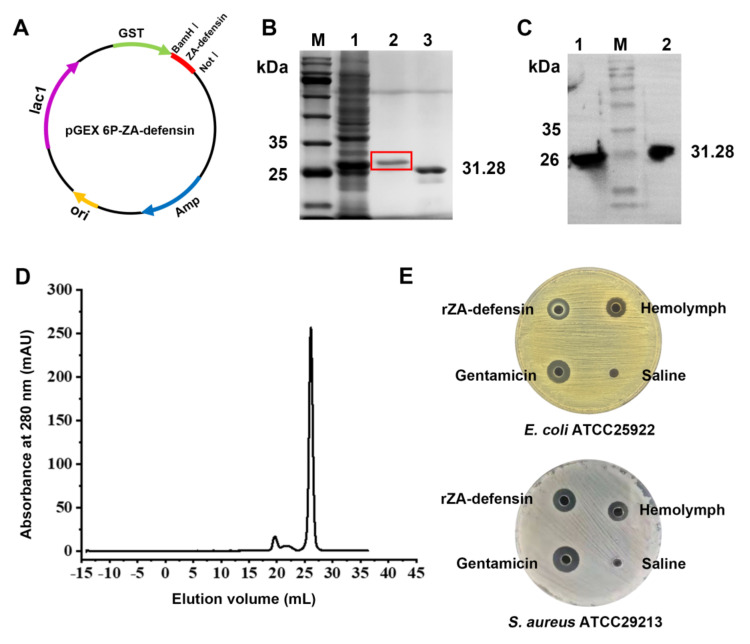
Expression and verification of recombinant *Z. atratus* defensin. (**A**) Construction of pGEX 6P-ZA-defensin. The gene codes mature *Z. atratus defensin* was cloned into pGEX 6P-1 with *BamH* I and *Not* I to construct recombinant plasmid pGEX 6P-ZA-defensin. (**B**) SDS-PAGE of recombinant ZA-defensin (rZA-defensin) expression. M: marker (10–180 kDa); 1: expression product of rZA-defensin induced by IPTG; 2: purified rZA-defensin; 3: GST-tag cut from rZA-defensin. (**C**) Western blotting analysis of purified rZA-defensin. 1: GST-tag cut from rZA-defensin defensin; M: marker (10–180 kDa); 2: purified rZA-defensin. (**D**) Purification of rZA-defensin by Superdex 30 after cutting off GST-tag shows a sharp peak at the elution volume of 26 mL. (**E**) rZA-defensin exhibits favorable antibacterial activity against *E. coli* ATCC25922 and *S. aureus* ATCC29213.

In this study, we amplified and analyzed the transcript of *Z. atratus defensin* (Figure S1), and then expressed mature *Z. atratus* defensin. As shown in Figure 1, recombinant *Z. atratus* defensin (rZA-defensin) could be identified by GST-antibody. After treating with Prescission Protease, there was only one sharp peak at the elution volume of 26 mL in Superdex 30. Mass spectrometry analysis of elution indicated that the sequence of rZA-defensin was entirely consistent with theoretical sequence (Figure S2). Furthermore, antimicrobial activity detection of rZA-defensin suggested that it had an observable antimicrobial activity against *Escherichia coli* ATCC25922 and *Staphylococcus aureus* ATCC29213.

### 2.2. Peptide Design and Structural Analysis

**Figure 2:**
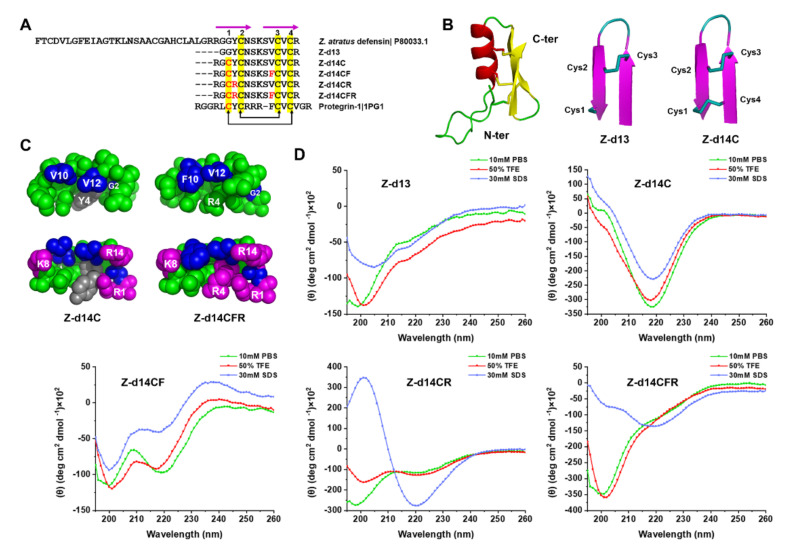
Design and structural analysis of *Z. atratus* defensin analogues. (**A**) Sequence alignment between *Z. atratus* defensin analogues and Protegrin-1 (PG-1, PDB ID: 1PG1). Rose lines represent the β-sheet region of peptides. The conserved cysteines are shadowed in yellow, the mutated residues are colored in red, the black arrows indicate the disulfide bonds of peptides. (**B**) Structural model (ribbon diagram) of *Z. atratus* defensin, Z-d13 and Z-d14C in accordance with (**A**). The structure of *Z. atratus* defensin was predicted according to Sapecin (PDB ID: 1L4V) by SWISS-MODEL (http://swissmodel.expasy.org/) (Accessed: 20 March 2022). *Z. atratus* defensin consists of *N*-terminal random coil followed by a α-helical and antiparallel β-sheet. Z-d14C forms a new disulfide bond between Cys1–Cys4 after mutating Gly into Cys at the 3rd position. (**C**) Clustering of hydrophilic residues (green), hydrophobic residues (blue) and positive residues (rose red) in Z-d14C and Z-d14CFR. (**D**) Circular dichroism (CD) spectra of *Z. atratus* defensin analogues in 10 mM PBS (green line), 50% trifluoroethanol (TFE) (red line) and 30 mM SDS (blue line).

Since ZA-defensin β-sheet shares similarity cysteine patterns with Progengrin-1 (PG-1, PDB ID: 1PG1), we remained the β-sheet region of ZA-defensin (Gly31 to Arg43), mutated Gly into Cys at the 3rd position so that the free sulfhydryl could be oxidized to form a new disulfide bond between Cys1 and Cys4 as same as PG-1, which further form a stable β-hairpin structure (Figure 2A). In order to increase the antimicrobial activity of peptide, we introduced Arg and Phe, respectively, to increase the net charges and hydrophobicity of peptide. As Table 1 showed, the net charges of Z-d13, Z-d14C, Z-d14CF, Z-d14CR, and Z-d14CFR were 2, 3, 3, 4 and 4. The retention time of Z-d13, Z-d14C, Z-d14CF, Z-d14CR, and Z-d14CFR was 9.077, 7.809, 9.538, 10.159 and 7.820, which indicated that the hydrophobicity of peptides follows the order Z-d14CR > Z-d14CF > Z-d13 > Z-d14CFR > Z-d14C. Structural prediction showed that the mutation of Gly at the 3rd position made Z-d14C, Z-d14CF, Z-d14CR and Z-d14CFR form a new intramolecular disulfide bond between Cys1 and Cys4, and the introduction of Arg and Phe made Z-d14CFR form a typical amphipathic structure with a hydrophobic patch and a cationic patch (Figure 2B,C).

To further investigate the secondary structural of peptides, we measured the far-UV CD spectra of each peptide in 10 mM phosphate-buffered saline (PBS), 50% 2,2,2-Trifluoroethanol (TFE) and 30 mM sodium dodecyl sulfate (SDS). Significantly, 10 mM PBS, 50% TFE, and 30 mM SDS were simulated hydrophilic environment, hydrophobic environment, and membrane-like environment, respectively. Our results showed that the CD spectrum of Z-d13 exhibited a single negative peak below 200 nm in 10 mM PBS, and a minimum around 205 nm and a crossover at 200 nm in 50% TFE and 30 mM SDS, which indicated Z-d14C formed a random coli in 10 mM PBS and formed a partial β-hairpin with one disulfide bond between Cys2 and Cys3 in 50% TFE and 30 mM SDS [17,25]. When we mutated Gly into Cys at the 3rd position, the CD spectrum of Z-d14C was characteristic of a minimum around 220 nm in 10 mM PBS, 50% TFE and 30 mM SDS, which suggested Z-d14C formed a conventional β-hairpin with two disulfide bonds between Cys1 and Cys4, Cys2 and Cys3 [25]. Additionally, when we mutated Val into Phe at the 9th position, Z-d14CF formed an unconventional β-hairpin, which was characterized by a negative ellipticity near 200 nm and 220 nm [26]. When Tyr was mutated into Arg at the 4th position, the CD spectrum of Z-d14CR exhibited a strong negative peak around 200 nm and a weak negative peak around 220 nm in 10 mM PBS and 50% TFE, and a strong positive peak around 200 nm and a negative peak around 220 nm in 30 mM SDS, which indicated Z-d14CR forms an unconventional β-hairpin in 10 mM PBS and 50% TFE, and a more stable conventional β-hairpin in 30 mM SDS. Furthermore, after we change Val and Tyr into Phe and Arg simultaneously, Z-d14CFR formed a partial β-hairpin in 10 mM PBS and 50% TFE, while formed a conventional β-hairpin in 30 mM SDS (Figure 2D) [17].

### 2.3. Functional Screening of ZA-Defensin Analogues

Antimicrobial ability was considered as an important index to screen out ZA-defensin analogues. Therefore, we detected the MIC values of each peptide to evaluate their antimicrobial ability. As shown in Table 2, Z-d13, Z-d14C and Z-d14CR displayed no antimicrobial activity at the concentration of 1 mg/mL. In contrast, Z-d14CF and Z-d14CFR exhibited enhanced antimicrobial activity than rZA-defensin. Among the peptides, Z-d14CFR exhibited potent antimicrobial activities against both Gram-positive and Gram-negative bacteria with the minimum inhibitory concentration (MIC) values ranged from 0.1 to 0.2 mg/mL. It also showed favorable antimicrobial ability to MDR clinical isolates (*E. coli*, *Klebsiella pneumoniae*, *Staphylococcus haemolyticus* and *Bacillus cereus*), but showed inactive to some bacteria, including *Proteus vulgaris*, *MRSA,* and *Streptococcus suis*.

**Figure 3:**
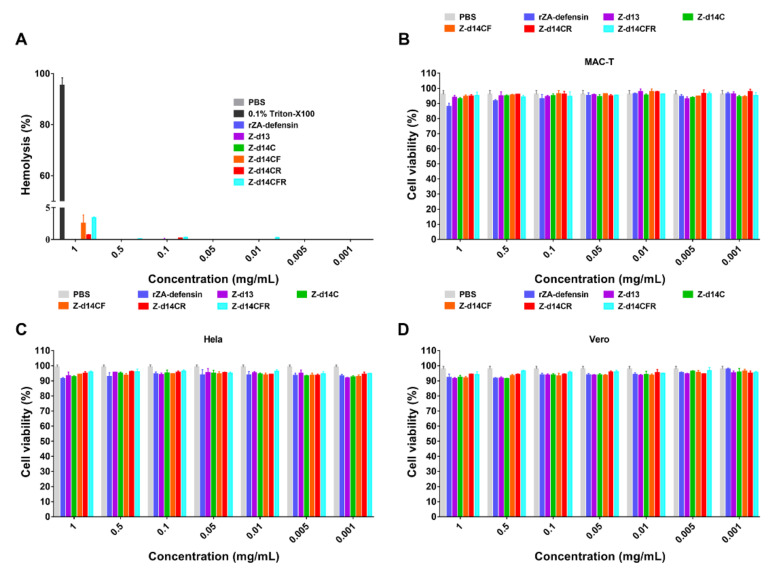
Hemolysis and cytotoxicity of rZA-defensin and its analogues. (**A**) Hemolysis of rZA-defensin defensin and its analogues. (**B**–**D**) Cytotoxicity of rZA-defensin defensin and its analogues to MAC-T (**B**), Hela (**C**), and Vero (**D**). The results are represented as mean ± SEM from three independent experiments.

Furthermore, cytotoxicity and hemolytic assays were carried out to evaluate the safety of peptides. The results showed that all the ZA-defensin analogues displayed less than 5% hemolysis and 10% cytotoxicity to mammalian cells even at the concentration as high as 1 mg/mL, suggesting ZA-defensin analogues have favorable safety (Figure 3).

After comprehensive consideration, Z-d14CFR was finally selected for the further study because of its favorable antimicrobial effect, low hemolysis and cytotoxicity.

### 2.4. Time-Killing Curves of Z-d14CFR

**Figure 4:**
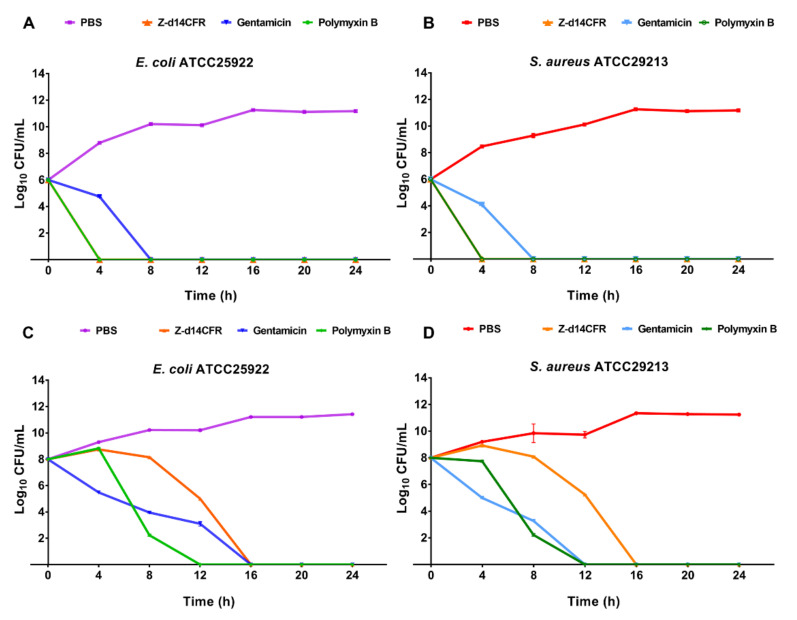
Time-killing curves of Z-d14CFR. Bacterial count of *E. coli* ATCC25922 (**A**) and *S. aureus* ATCC29213 (**B**) in logarithmic phase incubated with 2 MIC Z-d14CFR, polymyxin B or gentamicin. Bacterial count of *E. coli* ATCC25922 (**C**) and *S. aureus* ATCC29213 (**D**) in plateau incubated with 2 MIC Z-d14CFR, polymyxin B or gentamicin. The results are represented as mean ± SEM from three independent experiments.

In order to verify the lethality of Z-d14CFR to bacteria, we examined the bacteria-killing kinetics of Z-d14CFR (at a final concentration of 2 MIC) for *E. coli* ATCC25922 and *S. aureus* ATCC29213. As shown in Figure 4, Z-d14CFR and polymyxin B killed *E. coli* and *S. aureus* in the logarithmic phase within 4 h, while gentamicin killed the bacteria in the logarithmic phase within 8 h. In addition, Z-d14CFR killed *E. coli* and *S. aureus* in the plateau within 16 h, while polymyxin B and gentamicin killed the bacteria within 12 h. Followed that, we detected the MBC of Z-d14CFR, and found that Z-d14CFR at the concentration of 1 MIC could kill 99.9% bacteria. It suggested that Z-d14CFR was an efficient bactericidal agent as traditional antibiotics, rather than a growth-inhibiting agent (Table 2).

### 2.5. Z-d14CFR Destroyed Bacterial Membrane Integrity and Inhibited Biofilm Formation

**Figure 5:**
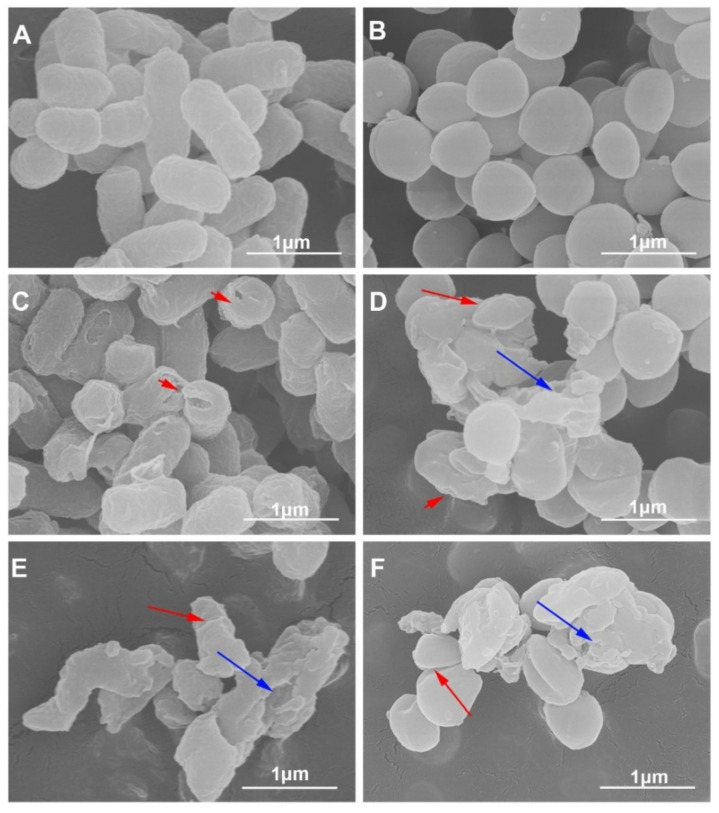
Morphological changes of bacteria treated with or without Z-d14CFR. Scanning electron microscopy (SEM) observation of *E. coli* ATCC25922 and *S. aureus* ATCC29213 treated with 10 mM PBS or 2 MIC Z-d14CFR for 3 h. *E. coli* ATCC25922 (**A**) and *S. aureus* ATCC29213 (**B**) treated with 10 mM PBS. *E. coli* ATCC25922 (**C**,**E**) and *S. aureus* ATCC29213 (**D**,**F**) treated with Z-d14CFR. The bacteria showed membrane breakage (red short arrows), cells shrinkage (red long arrows) and rupture (blue long arrows).

Scanning electron microscopy was used to observe the direct effect of Z-d14CFR on bacterial ultrastructure. As shown in Figure 5, the normal *E. coli* and *S. aureus* cells had a plump shape with intact membranes (Figure 5A,B). After incubating with Z-d14CFR for 3 h, the bacteria appeared severe damage, including membrane breakage, cells shrinkage and rupture (Figure 5C–F). Thus, we speculated that Z-d14CFR killed bacteria via disrupting the integrity of bacterial cell membranes.

**Figure 6:**
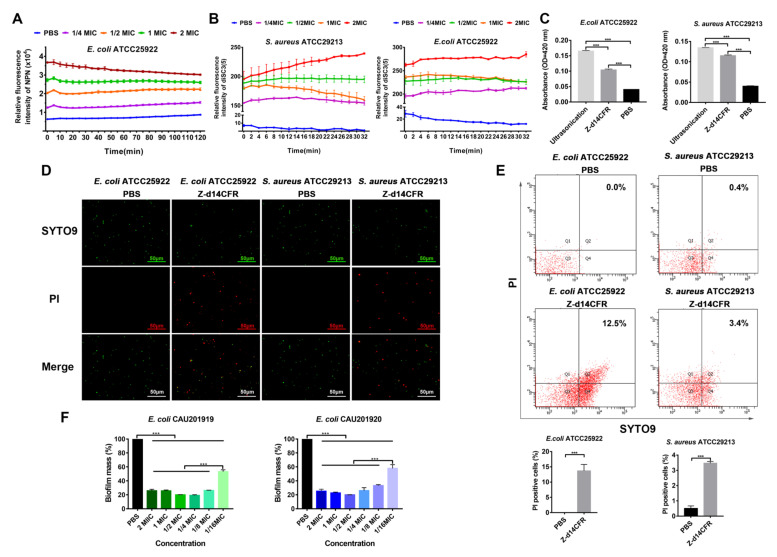
Antibacterial mechanism of Z-d14CFR. (**A**) Dynamic curves of NPN uptake in *E. coli* ATCC25922 after incubating with 2 MIC, 1 MIC, 1/2 MIC, 1/4 MIC Z-d14CFR, 10 mM PBS as the negative control. (**B**) Dynamic curves of DiSC_3_(5) release in *E. coli* ATCC25922 and *S. aureus* ATCC29213 after incubating with 2 MIC, 1 MIC, 1/2 MIC, 1/4 MIC Z-d14CFR, 10 mM PBS as the negative control. (**C**) Extracellular release of β-galactosidase in *E. coli* ATCC25922 and *S. aureus* ATCC29213 induced by 2 MIC Z-d14CFR, 10 mM PBS as the negative control, ultrasonication as the positive control. (**D**) SYTO9/PI staining of *E. coli* ATCC25922 and *S. aureus* ATCC29213 treated with 10 mM PBS or 2 MIC Z-d14CFR for 3 h under laser confocal microscope. (**E**) Flow cytometry of PI-positive cells treated with 10 mM PBS or 2 MIC Z-d14CFR for 3 h. (**F**) Biofilm formation of *E. coli* CAU201919 and *E. coli* CAU201920 treated with 2 MIC, 1 MIC, 1/2 MIC, 1/4 MIC, 1/8 MIC and 1/16 MIC Z-d14CFR or 10 mM PBS. All the results are represented as mean ± SEM from three independent experiments, *** *p* < 0.001, unpaired *t*-test.

In order to further investigate the effect of Z-d14CFR on membranes, we first measured the hydrophobic fluorophore NPN incorporated into the outer membrane barrier of *E. coli*. As Figure 6A showed, Z-d14CFR significantly increased NPN uptake of *E. coli* ATCC25922 in a concentration-dependent fashion, indicating Z-d14CFR permeabilized the outer membrane of *E. coli*.

Subsequently, the effect of Z-d14CFR on electrochemical potential disruption across the bacterial cytoplasmic membrane was detected by DiSC_3_(5), a membrane potential-associated cationic probe. As shown in Figure 6B, the addition of Z-d14CFR resulted in remarkable releasing of DiSC_3_(5), which suggested Z-d14CFR triggered off the depolarization of cytoplasmic membrane.

Followed that, the release of β-galactosidase was detected to assess the permeabilization of Z-d14CFR on cytoplasmic membrane. As expected, compared with PBS, Z-d14CFR significantly induced the release of β-galactosidase in *E. coli* ATCC25922 and *S. aureus* ATCC29213 to the outside of cells (*p* < 0.001) (Figure 6C), which proved that Z-d14CFR enhanced the permeability of cytoplasmic membrane.

SYTO 9/PI staining was carried out to further study the effects of Z-d14CFR on both membrane integrity and viability of bacteria. SYTO 9 generally penetrates bacteria with intact membranes, whereas PI only penetrates bacteria with damaged membranes. Therefore, bacteria with intact membranes usually stain fluorescent green by SYTO 9, whereas bacteria with damaged membranes stain fluorescent red by PI. Laser confocal microscopy observations showed that nearly all *E. coli* and *S. aureus* treated with PBS were stained green, and only few bacteria were stained red. In contrary, after treating with Z-d14CFR, the number of red bacteria (PI-positive) in both *E. coli* and *S. aureus* was significantly increased (Figure 6D).

Furthermore, flow cytometry was used to detect the population of PI-positive bacteria and SYTO 9-positive bacteria in different treatment groups. As shown in Figure 6E, PBS induced 0% *E. coli* ATCC25922 and 0.4% *S. aureus* ATCC29213 to be PI-positive, while Z-d14CFR, respectively, induced 12.5% *E. coli* ATCC25922 and 3.4% *S. aureus* ATCC29213 to be PI-positive, which indicated that Z-d14CFR possessed a strong effect on bacterial membrane permeabilization.

Biofilm formation promotes bacterial pathogenicity and drug-resistance development [27,28]. Therefore, we measured the influence of Z-d14CFR on bacterial biofilm formation. As shown in Figure 6F, Z-d14CFR obviously suppressed biofilm formation, and a final concentration of 1/16 MIC Z-d14CFR could inhibit nearly 50% biofilm formation.

### 2.6. Stability of Z-d14CFR

The stability of Z-d14CFR was tested by MIC changes in different treatments. As shown in Table 3, at the conditions of 37–100 °C, pH = 4–8 and UV exposure, the MIC values of Z-d14CFR against *E. coli* ATCC25922 and *S. aureus* ATCC29213 had no change, and at the conditions of 121 °C, extreme pH values (pH < 4 or pH > 9) and 10% FBS, the antimicrobial activities of Z-d14CFR against *E. coli* and *S. aureus* was slightly decreased. However, after incubating with pepsin, trypsin, papain, α-chymotrypsin or proteinase K, Z-d14CFR became inactive to bacteria even at a high concentration of 2 mg/mL, which indicated that proteinase could completely eliminate the antimicrobial activity of Z-d14CFR.

### 2.7. Z-d14CFR Enhanced Bacterial Clearance in a Murine Model of Mastitis

**Figure 7:**
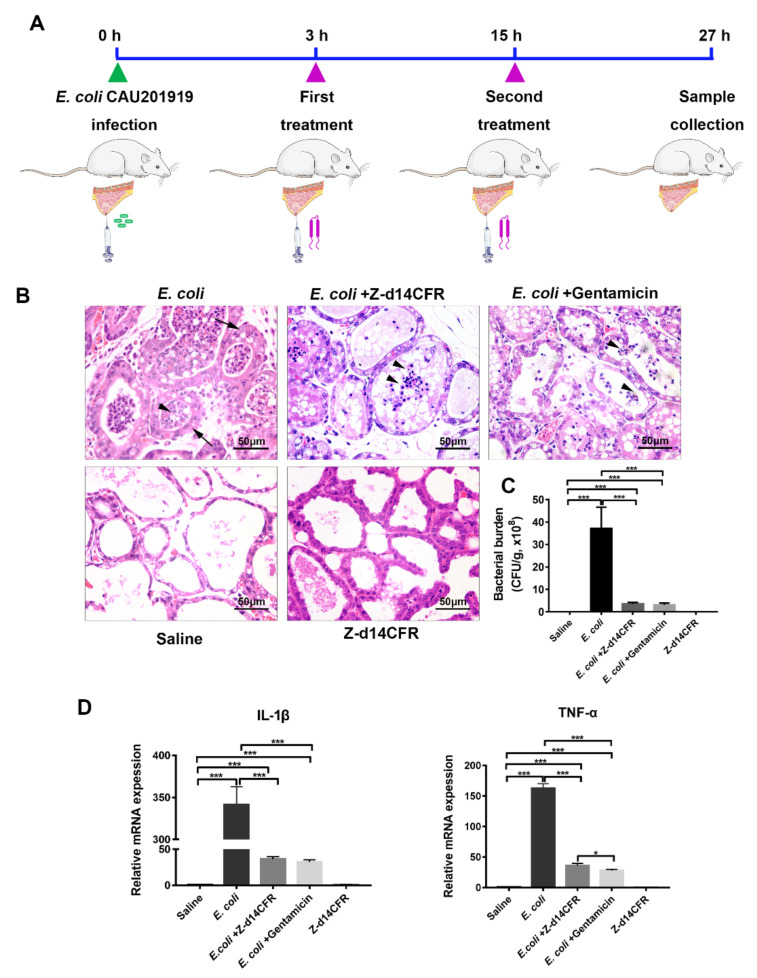
A murine model of multidrug-resistant *E. coli*-induced mastitis treated with or without Z-d14CFR. (**A**) Experimental protocol of murine mastitis model induced by multidrug-resistant *E. coli* CAU201919. (**B**) Histopathologic changes of the mammary gland. Long arrows indicate the thickening of mammary acinar walls, short arrows indicate neutrophils infiltration in acinar cavity. (**C**) Bacterial burden in the mammary gland of mice. (**D**) *IL-1 β* and *TNF-α* mRNA expression in the mammary gland of mice. The results are represented as mean ± SEM from three independent experiments, * *p* < 0.05, *** *p* < 0.001, unpaired *t*-test.

To explore the potential of Z-d14CFR application, we constructed a murine model of mastitis induced by MDR *E. coli* CAU201919 and assessed the therapeutic effects of Z-d14CFR on MDR bacterial infection in vivo (Figure 7A).

At 27 h post-infection, the mice exhibited severe mastitis, including thickening of the acinar walls and neutrophils infiltrated in the acinar cavity of the mammary gland, whereas the damage was obviously ameliorated when treated with Z-d14CFR or gentamicin, which mainly manifested by recovery of acinar walls thickening and decreased neutrophils infiltration (Figure 7B). Consistently, bacterial burden in the mammary gland of mice infected only with *E. coli* was about 3.71 × 10^9^ CFU/g tissue, while it was reduced by either Z-d14CFR or gentamicin treatment (*p* < 0.001), which the number of *E. coli* in the mammary gland declined to 3.57 × 10^8^ and 3.13 × 10^8^ CFU/g tissue, respectively (Figure 7C). Furthermore, *E. coli* infection resulted in an increase in the mRNA expression of the pro-inflammatory factors TNF-α and IL-1β in the mammary gland, but this increase was attenuated by either Z-d14CFR or gentamicin treatment (*p* < 0.001) (Figure 7D).

## 3. Discussion

**Figure 8:**
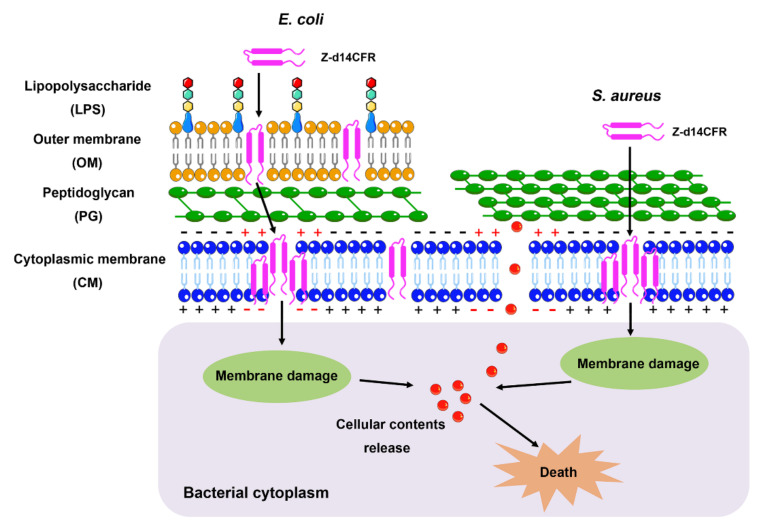
Possible mechanism of Z-d14CFR against *E. coli* and *S. aureus*. Z-d14CFR binds to bacterial membranes through electrostatic interactions, induces cytoplasmic membrane depolarization, inserts into the cell membranes and forms β-barrels, thereby disrupting the integrity of cell membranes, promoting the release of cellular contents and then killing the bacteria.

In this study, we successfully amplified the transcript of *Z. atratus defensin*, and expressed recombinant *Z. atratus* defensin (rZA-defensin) with favorable antimicrobial activity against *E. coli* ATCC25922 and *S. aureus* ATCC29213. Unfortunately, rZA-defensin only exhibited antimicrobial activity after cutting off the GST-tag, which undoubtedly increased the process and cost of ZA-defensin production. To further obtain antimicrobial peptides with great antimicrobial activity and commercial-scale production, we transformed ZA-defensin and looked forward to obtaining optimal ZA-defensin analogues. Interestingly, we found that Z-d14CFR could induce bacterial death by destroying bacterial membranes and promoting cellular contents release (Figure 8).

Similar to most insect defensins, mature ZA-defensin is composed of a n-loop followed by a compact α-helical and β-sheet. Interestingly, the region of ZA-defensin β-sheet can be well superimposed with some natural β-hairpin AMPs, which exhibited high activity against pathogens [24,26,29]. The peptides derived from the region of insect defensin β-sheet have been proved to possess antibacterial activity [17,19]. In this study, we synthesized the region of ZA-defensins β-sheet with an amidated C-terminus (Z-d13) and found that Z-d13 had a slight activity against bacteria at a high concentration of 3 mg/mL (data not shown). In order to improve the antimicrobial activity of Z-d13, we mutated Gly into Cys at the 3rd position, and hoping to form a new intramolecular disulfide bond between Cys1 and Cys4, because Z-d13 shared conserved cysteines with some natural β-hairpin AMPs, for example PG-1 [30]. The CD spectrum of Z-d14C revealed that there was a new intramolecular disulfide bond formation in Z-d14C. However, Z-d14C still showed weak activity against bacteria as same as Z-d13, which indicated that intramolecular disulfide bonds are not the key factor for antimicrobial effect of Z-d14C.

Subsequently, we introduced Arg and Phe to improve the amphiphilicity and net charges of peptides. Our results showed that Z-d14CFR exhibited the highest antimicrobial activity, even though Z-d14CR had the highest positive charges and hydrophobicity. In general, positive charge and hydrophobic amino acids are the key parameters for AMPs [31,32]. The positive charge of AMPs mainly relies on the number of cationic residues, including arginine (Arg), lysine (Lys), and histidine (His). They usually interact with the negatively charged components of bacterial membrane, such as lipopolysaccharide (LPS), lipoteichoic acid (LTA), and phosphatidyl glycerol (PG), which resulted in the high accumulation of bacterial membranes, therefore enhancing the membrane permeability and bactericidal activity [9,33]. Moreover, hydrophobicity could control the depth of peptides inserted into bacterial membranes. Adding the hydrophobicity of peptides makes it easier to reach the threshold of membrane rupture, thereby further inducing strong antimicrobial activity [34,35]. Structural analysis of peptides found that the phenyl ring of Phe increased the hydrophobic surface of Z-d14CF and Z-d14CFR, which might make them easier to bind to cell membranes, thereby exerting higher antimicrobial effect. Furthermore, Z-d14CFR has a typical amphiphilic structure with a Arg-rich cationic domain and a hydrophobic domain. It was reported that Arg could form a strong hydrogen bond with phosphate of lipid bilayer, thus promoting the deeper insertion of the membrane and giving AMPs a stronger membrane-disrupting ability [36,37]. It may be the main reason for Z-d14CFR to perform the higher antimicrobial effect than other peptides.

Since Z-d14CFR has the optimal antimicrobial effect, it was chosen for the further research. Time-killing curves revealed that Z-d14CFR had the same bactericidal effect as traditional antibiotics. Subsequently, the antimicrobial mechanism of Z-d14CFR was investigated. SEM observation revealed that Z-d14CFR destructed the membranes of bacteria. The results of NPN uptake, DiSC_3_(5) assay and SYTO 9/PI staining further proved Z-d14CFR performed antimicrobial effect via targeting bacterial membranes. The release of β-galactosidase in *E. coli* and *S. aureus*, on the other hand, suggested that Z-d14CFR killed bacteria through inducing cellular contents release. Prior study demonstrated that peptides with β-hairpin could form transmembrane β-barrels in biological membranes which in turn increase membrane disruption and induce bacterial death. It was showed that d14CFR formed a stable β-hairpin in membrane-like (30 mM SDS) environment. Therefore, we presumed that Z-d14CFR could insert into the cell membranes and form β-barrels, thereby promoting the release of cellular content and killing bacteria. In addition, our study also found that 1/16 MIC Z-d14CFR could inhibit nearly 50% biofilm formation. Biofilms are surface-associated bacterial communities that play an important role in antibiotic resistance, and disruption of biofilms makes bacteria more sensitive to antimicrobial agents [38]. Furthermore, Z-d14CFR is effective to both Gram-positive bacteria and Gram-negative bacteria, including multidrug-resistant clinical isolates, which indicated that Z-d14CFR targets bacterial membranes in a nonspecific manner. This interaction pattern prompts Z-d14CFR has a broad-spectrum antimicrobial activity and extensive applications.

Hemolysis and cytotoxicity are mainly bottlenecks affecting clinical application of AMPs. Generally, the hemolysis and cytotoxicity of AMPs will enhance with the increase of hydrophobicity and positive charge [39]. In our study, all the peptides exhibited less than 5% hemolysis and 10% cytotoxicity of mammalian cells, indicating that ZA-defensin analogues did not bind to mammalian cell membrane. In fact, the membrane of mammalian cells is usually composed of zwitterionic phospholipids (for example phosphatidylcholines, PC) while bacterial cell membrane is usually composed of a large amount of negatively charged phospholipids, such as LPS, LTA, PG et al. Normally, the cationic AMPs selectively bind to negative charges of bacterial membranes, and show inactive to mammalian cells [40,41]. Therefore, our results, on the other hand, illustrated that Z-d14CFR targeted bacterial membranes through interacting with negative charges.

In addition, stability directly determines the potential clinical application of AMPs. In most case, AMPs would decrease or even lose antimicrobial activity in the condition of proteases, serum, UV exposure, extreme temperature and pH value. To determine the stability of Z-d14CFR, we measured the MIC values of Z-d14CFR against *E. coli* ATCC25922 and *S. aureus* ATCC29213 in different conditions. Encouragingly, Z-d14CFR kept favorable antimicrobial activity in the condition of UV exposure, 10% FBS, extreme temperature (37–121 °C) and extreme pH value (pH = 2–11), which suggested that Z-d14CFR might have extensive applications in many processes of production and preservation, such as medicine and medical instruments. Significantly, the proteases, including pepsin, trypsin, papain, α-chymotrypsin, and proteinase K, completely destroyed the antimicrobial activity of Z-d14CFR, which suggested that it was necessary for Z-d14CFR to choose intramuscular injection or transdermal administration instead of oral administration, when it was used as a drug in clinic. Low protease stability is an inevitable issue for AMPs in clinical applications [42], thus, the future optimizations of Z-d14CFR need to focus on resisting enzymolysis.

As expected, we also evaluated the therapeutic potential of Z-d14CFR on multidrug-resistant bacterial infection in vivo based on its favorable bactericidal activity and safety in vitro. Our results showed that Z-d14CFR enhanced bacterial clearance and reduced neutrophils infiltration and pro-inflammatory cytokines (TNF-α, IL-1β) expression, hence alleviated *E. coli*-induced inflammation in a murine model of mastitis. Our findings suggested that Z-d14CFR might be a promising antimicrobial candidate for facilitating the resolution of MDR bacterial infection. Our study just preliminarily evaluated the biological activity of Z-d14CFR in vitro/vivo, the application (for example in combination with existing antibiotics) and route of administration of Z-d14CFR in the treatment of MDR bacterial infection still need to be further explored.

## 4. Materials and Methods

### 4.1. Ethics Statement

In this study, all animals were treated strictly in accordance with the Guidelines for Laboratory Animal Use and Care from the Chinese Center for Disease Control and Prevention and the Rules for Medical Laboratory Animals from the Chinese Ministry of Health, under protocol AW03111202-1-3 approved by the Animal Ethics Committee of China Agricultural University. All the pathogens were used strictly according to the Regulations on Biological Safety Management of Pathogen Microbiology Laboratory (000014349/2004-00195) from the State Council of the People’s Republic of China.

### 4.2. Bacterial Strains and Cell Lines

*E. coli* ATCC25922, *E. coli* CVCC1450, *S. aureus* ATCC29213, *S. typhimurium* ATCC14028, *MRSA* ATCC33591, *P. vulgaris* CVCC1971, *S. suis* CVCC3307 were purchased from China Institute of Veterinary Drug Center (Beijing, China). *E. coli* CAU201919, *E. coli* CAU201920, *K. pneumoniae* CAU202084, *S. haemolyticus* CAU202078, *B. cereus* CAU 202020 were isolated from unpasteurized milk and preserved in the College of Veterinary Medicine, China Agricultural University (Beijing, China). The information of strains was listed in Table S1. Hela and Vero cells were preserved in the College of Veterinary Medicine, China Agricultural University (Beijing, China). Bovine mammary alveolar (MAC-T) cells were gifted by Dr. Ying Yu from China Agricultural University.

### 4.3. Gene Amplification and Recombinant Protein Expression

The conserved sequence of *Z. atratus defensin* was amplified by PCR with degenerate primers (5′-AAACGCTTYACWTGTGACGTWCT-3′ and 5′-TCATTAKCGRCAAACGCARAC-3′). Then 5′ and 3′ of *Z. atratus defensin* transcript were obtained by 5′/3′ RACE PCR according to the manufacturer’s instructions of SMARTer RACE 5′/3′ Kit (Takara, Dalian, China). The primers were 5′-TGGTTTCGAAATTGCAGGAACAAAACTGAATAGCG-3′ and 5′-AGATTTAGAGTTACAATAACCCCCTCTCCTCCCT-3′for 5′ RACE PCR, and 5′-TGCTTGTGGTGCTCATTGTCTAG-3′ and 5′-ACAATAACCCCCTCTCCTCCCTA-3′ for 3′ RACE PCR. The homology of *Z. atratus* defensin was compared by GenBank protein database. The three-dimensional structure was built from Sapecin (PDB ID: IL4V) online with SWISS-MODEL (https://swissmodel.expasy.org/) (Accessed: 20 March 2022). The 208-339 bases of *Z. atratus* defensin were amplified by PCR with the following primers, 5′-CGGGATCCTTCACCTGCGACGTTCTTG-3′ and 5′-TAAAGCGGCCGCTTAACGACATACACAAACAGATTTAGAGT-3′. Then, the fragment was digested with *BamH* I and *Not* I (Takara, Dalian, China), cloned into pGEX 6P-1, and transformed into *E. coli* Rosetta (DE3) cells (Solarbio, Beijing, China). The recombinant protein expression was induced with 0.8 mM isopropy1-β-D-1-thiogalactopyranoside (IPTG) (Solarbio, Beijing, China) at 16 °C for 20 h, purified through GSTrap 4B (Cytiva, Marlborough, MA, USA) and treated with Prescission Protease (Cytiva, Marlborough, MA, USA) at 4 °C for 16 h to remove glutathione S-transferase (GST)-tag. Then, the rZA-defensin and GST-tag were verified by Western blotting with GST Antibody (B-14) (1:1000, Sc-138, Santa, Dallas, TX, USA). Finally, the target protein was further purified by SuperdexTM 30 (Cytiva, Marlborough, MA, USA), and the sequence of recombinant *Z. atratus* defensin were determined by mass spectrometry.

### 4.4. Peptide Design and Synthesis

The peptides derived from *Z. atratus* β-sheet defensin were chemically synthesized by Sangon Biotech. Co., Ltd. (Shanghai, China). The structures of peptides were predicted according to Protegrin-1 (PG-1, PDB ID: 1PG1) by Modeller and further modified by PyMOL.

### 4.5. Circular Dichroism (CD) Spectroscopy

The CD spectrum of peptides in 10 mM PBS, 50% TFE (*v*/*v*), and 30 mM SDS was measured by Chirascan^TM^-plus Circular Dichroism Spectrometer (Applied Photophysics Ltd., London, UK) using a 0.5 mm quartz cuvette. An average of three scans was collected from 195 nm to 260 nm at 100 nm/min. Data are expressed as mean residue molar ellipticity.

### 4.6. Antimicrobial Activity

The MIC of peptides against bacteria was carried out by the microdilution broth method. The bacterial culture (OD 600 = 0.6) was diluted in 10 mM PBS to 1 × 10^6^ CFU/mL. The bacterial suspension (100 μL) was added to 96-well plates, and the same volume of peptides, ampicillin or gentamicin (concentrations ranging from 0.001 to 2 mg/mL) were then added to the same 96-well plates, respectively. The cells were incubated for 18 h at 37 °C and the MIC of peptides was determined as the minimum concentration that completely inhibits the growth of bacteria, and the minimum concentration which kills 99.9% bacteria was regard as the MBC.

### 4.7. Hemolysis

Briefly, fresh sheep blood (Solarbio, Beijing, China) was centrifuged at 3000× *g* for 10 min at 4 °C and washed thrice with 10 mM PBS to isolate red blood cells (RBC). The peptides were dissolved into 10 mM PBS and diluted to a concentration ranging from 2 mg/mL to 0.002 mg/mL. Then, 100 μL RBC (8%, *v*/*v*) was incubated with 100 μL peptide at 37 °C for 1 h. 10 mM PBS and 0.2% Triton X-100 were, respectively, used as negative control and positive control [43]. The absorption of released hemoglobin was measured at 570 nm with a microplate reader (Thermo, Waltham, MA, USA). The hemolysis rate was calculated as follows: Hemolysis (%) = (OD 570 peptide − OD 570 PBS)/(OD 570 Triton X-100 − OD 570 PBS) × 100%.

### 4.8. Cytotoxicity

The cytotoxicity of peptides to mammalian cells was carried out according to CCK8 instructions (Beyotime, Shanghai, China). 1 × 10^4^ Hela, Vero and MAC-T cells were, respectively, added in 96-well plates and cultured at 37 °C with 5% CO_2_ for 24 h [43]. Then, the cells were washed thrice with 10 mM PBS to remove medium and then incubated with 100 μL peptide in DMEM with 1 % (*v*/*v*) FBS (finally concentration ranging from 1 mg/mL to 0.001 mg/mL). After incubating at 37 °C with 5% CO_2_ for 24 h, 10 μL CCK8 was added to the cells and then continued to be incubated at 37 °C for 4 h. CCK8 interacted with PBS without cells as the blank control. The absorbance of the mixture was measured at 450 nm and cell viability was calculated as follows: Cell viability (%) = (OD 450 peptide − OD 450 Blank)/(OD 450 PBS − OD 450 Blank) × 100%.

### 4.9. Time-Killing Assays

*E. coli* ATCC25922 and *S. aureus* ATCC29213 in the mid-logarithmic phase were diluted into 1 × 10^6^ CFU/mL with 10 mM PBS, and incubated with 10 μL Z-d14CFR in a final concentration of 2 MIC. At 0, 4, 8, 12, 16, 20, and 24 h, the mixture was serial diluted and spread onto LB agar plates [23]. After 16 h of cultivation, the CFU number of bacteria was counted to evaluate the ability of peptide to kill bacteria. The bacteria treated with 10 mM PBS as a negative control, and meanwhile incubated with 2 MIC gentamicin or polymyxin B as a positive control.

### 4.10. Stability Assessment

The stability of Z-d14CFR was measured according to our previous study [23]. Briefly, peptide was treated with different temperature (37 °C, 60 °C, 80 °C, 100 °C and 121 °C), UV exposure, or incubated with a buffer in different pH values (pH = 2–12), 10% fetal bovine serum (FBS) and protease (pepsin, trypsin, papain, α-chymotrypsin and proteinase K). Subsequently, the changes in antimicrobial activity of peptides were measured to evaluate the stability of peptides. *E. coli* ATCC25922 and *S. aureus* ATCC29213 were the indicative strain for the above experiment.

### 4.11. Scanning Electron Microscopy (SEM)

*E. coli* ATCC25922 and *S. aureus* ATCC29213 (OD 600 = 0.6) were washed with 10 mM PBS, and then treated with Z-d14CFR in a final concentration of 2 MIC at 37 °C for 3 h. Then the cells were washed 3 times with 10 mM PBS, followed by fixing with 3% glutaraldehyde, dehydrating with a graded series of ethanol and drying. Finally, the cells were sputtered with gold coating and observed with a scanning election microscope (SU-8010, Hitachi, Japan).

### 4.12. Biofilm Inhibition Assays

The ability of Z-d14CFR affect bacterial biofilm formation was analyzed by crystal violet (Solarbio, Beijing, China) stain. Multidrug-resistant *E. coli* strain CAU201919 and CAU201920 with strong biofilm formation ability were used in this experiment. Briefly, 190 μL 1 × 10^6^ CFU/mL *E. coli* was incubated with 10 μL Z-d14CFR at a final concentration of 2 MIC, 1 MIC, 1/2 MIC, 1/4 MIC, 1/8 MIC and 1/16 MIC at 37 °C for 36 h in LB medium. Afterwards, the planktonic bacteria were discarded and washed with 10 mM PBS 3 times. The biofilms were then fixed with 100% methanol for 10 min and stained with 1% crystal violet for 15 min. After washing with 10 mM PBS 3 times, 30% acetic acid was added to dissolve the crystal violet fixation, and the absorbance value was recorded at 570 nm. Percentage biofilm inhibition was calculated as follows: Biofilm mass (%) = (ODZ-d14CFR/ODPBS) × 100%.

### 4.13. Outer Membrane Permeability

The changes in outer membrane permeability of *E. coli* caused by Z-d14CFR were measured by the fluorescent dye N-phenyl-1-napthylamine, NPN (Macklin, N816201, Shanghai, China). *E. coli* ATCC25922 cells (OD 600 = 0.5) were washed and diluted into 5 mM HEPES buffer (containing 5 mM glucose, pH 7.4) and then incubated with 10 μM NPN in the dark at 37 °C for 30 min. 10 μL Z-d14CFR in a final concentration of 2 MIC, 1 MIC, 1/2 MIC, and 1/4 MIC was added and the fluorescence intensity within 60 min was continuously recorded on a microplate reader (M200, Tecan, Infinite) at a 350 nm excitation wavelength and a 420 nm emission wavelength.

### 4.14. Cytoplasmic Membrane Depolarization

The ability of Z-d14CFR to disrupt inner cytoplasmic membrane potential of *E. coli* ATCC25922 and *S. aureus* ATCC29213 was monitored using 3,3-dipropylthiadicarbocyanine iodide DiSC_3_(5) (Macklin, D864435, Shanghai, China) as previous mentioned [43]. The bacteria were washed and resuspended with 5 mM HEPES, pH 7.4, containing 5 mM glucose and 100 mM KCl buffer into 1 × 10^6^ CFU/mL. DiSC_3_(5) was subsequently added and incubated at 37 °C in the dark for 15 min until the fluorescence had stabilized. Z-d14CFR in a final concentration of 2 MIC, 1 MIC, 1/2 MIC and 1/4 MIC was added to the mixture. The fluorescence intensity was continuously monitored for 30 min by an excitation wavelength of 622 nm and an emission wavelength of 670 nm.

### 4.15. Cytoplasmic Membrane Integrity

*E. coli* ATCC25922 and *S. aureus* ATCC29213 (OD600 = 0.6) were washed and diluted into 1 × 10^8^ CFU/mL in sterile saline. The cells were treated with Z-d14CFR in the final concentration of 2 MIC for 3 h. Following that, the same volume of a mixture contains SYTO 9 dye and PI (Thermo Fisher, Waltham, MA, USA) (*v*/*v*, 1:1) was added to bacteria and incubated at 37 °C in the dark for 15 min. The bacteria were washed with saline to remove the unbound dye and then observed with a Nikon A1 confocal laser scanning microscope.

In order to further determine the percentage of PI-positive cells, the data of 10,000 cells were collected and analyzed by BD LSRFortessa flow cytometer.

### 4.16. Extracellular β-Galactosidase Assay

*E. coli* ATCC25922 and *S. aureus* ATCC29213 were washed and resuspended with 10 mM PBS into OD 600 = 0.5. The cells were then treated with Z-d14CFR in a final concentration of 2 MIC for 4 h at 37 °C and centrifuged at 12,000× *g* at 4 °C for 10 min to collect supernatant. Then, 190 μL of the supernatants was incubated with 10 μL 2-nitrophenyl-β-d-galactopyranoside, ONPG (Macklin, N814742, Shanghai, China) at a final concentration of 3 mM at 37 °C for 30 min. The absorbance at 420 nm was measured to assess the level of β-galactosidase outside the cells. The bacteria treated with 10 mM PBS in absence of peptide as the negative control and treated with ultrasonication as the positive control.

### 4.17. Mouse Infection Models

In this study, the therapeutic effects of Z-d14CFR on MDR bacterial infection in vivo was verified by a murine mastitis model. *E. coli* CAU201919 was used to establish the mastitis model because it is highly pathogenic compared with other bacteria and is resistant to various antibiotics, such as ampicillin, amoxicillin and cefazolin. In brief, 45 Crl:CD1 female mice at 17–18 days of gestation were purchased from Beijing Vital River Laboratory Animal Technology Co., Ltd. (Beijing, China) and kept in the Laboratory Animal Center of China Agricultural University (Beijing, China). At 72 h post-partum, the mice were randomly divided into 5 groups (*n* = 9 per group) and the offspring were removed 3 h before of administration. The mice were anesthetized by Zoletil 50 (55 mg/kg body weight, WK001, Virbac, France) and held under the stereoscope in a supine position. Mice in the saline group and Z-d14CFR group were intraductal inoculated with 50 μL normal saline or 50 μL Z-d14CFR (25 μg each teat) alone to the milk duct. Mice in other groups were inoculated with 25 μL (1 × 10^5^ CFU) *E. coli* CAU201919 suspension to the milk duct, and after 3 h of *E. coli* challenged, mice were treated with 25 μL saline, Z-d14CFR (25 μg) or gentamicin (10 μg), respectively, via milk duct injection. Then the mice were given the same treatment after 15 h of *E. coli* challenged. After 27 h of *E. coli* challenged, all mice were euthanized. The mammary gland tissues were collected to carry out bacterial count, histopathological examination and inflammatory factors examination.

### 4.18. Statistical Analysis

All statistical analysis was performed using GraphPad Prism 7 with unpaired *t*-test with Bonferroni correction. Data were presented as means ± SEM. *p* < 0.05 was considered statistically significant.

## 5. Conclusions

In this study, we successfully expressed rZA-defensin and designed a Arg-rich β-hairpin peptide Z-d14CFR with increased antimicrobial activity based on the structural similarity between ZA-defensin β-sheet and natural β-hairpin, protegrin-1. According to our results, Z-d14CFR possesses suitable safety and stability in vitro, and can kill bacteria via destroying bacterial membranes and promoting cellular contents release. In the murine model of mastitis, Z-d14CFR exhibits favorable therapeutic potential by enhancing the clearance of *E. coli* in the mammary gland, reducing neutrophils infiltration and expression of TNF-α and IL-1β. In summary, Z-d14CFR may be a potential candidate for antibiotics alternatives against multidrug-resistant bacteria.

## Figures and Tables

**Table 1:** Peptide information.

Peptide	Sequence	Formula	MW ^a^	Net Charge (+)	PI	RT (min) ^b^
Z-d13	GGYCNSKSVCVCR-NH_2_	C_54_H_91_N_19_O_17_S_3_	1374.6	2	8.68	9.077
Z-d14C	RGCYCNSKSVCVCR-NH_2_	C_61_H_105_N_23_O_18_S_4_	1576.88	3	8.94	7.809
Z-d14CF	RGCYCNSKSFCVCR-NH_2_	C_65_H_105_N_23_O_18_S_4_	1624.92	3	8.94	9.538
Z-d14CR	RGCRCNSKSVCVCR-NH_2_	C_58_H_108_N_26_O_17_S_4_	1569.89	4	9.38	10.159
Z-d14CFR	RGCRCNSKSFCVCR-NH_2_	C_62_H_108_N_26_O_17_S_4_	1617.94	4	9.38	7.820

^a^ Molecular weight (MW) measured by MALDI-TOF MS; ^b^ RT: Retention time (RT) of peptide in HPLC.

**Table 2:** MIC (MBC) ^a^ of Z-d14CFR against bacteria (mg/mL).

Bacteria	rZA-Defensin	Z-d13	Z-d14C	Z-d14CF	Z-d14CR	Z-d14CFR	Ampicillin	Gentamicin
*Escherichia coli* ATCC25922	0.5 (0.5)	NA (NA) ^c^	0.8 (0.8)	0.4 (0.4)	0.8 (0.8)	0.1 (0.1)	0.004 (0.016)	0.004 (0.004)
*Escherichia coli* CVCC1450	0.5 (0.5)	NA (NA)	NA (NA)	0.4 (0.4)	0.8 (0.8)	0.1 (0.1)	0.008 (0.032)	0.002 (0.004)
*Escherichia coli* CAU 201919	0.5 (0.5)	NA (NA)	NA (NA)	0.4 (0.4)	0.8 (0.8)	0.1 (0.1)	NA (NA)	0.04 (0.04)
*Escherichia coli* CAU 201920	0.5 (0.5)	NA (NA)	0.8 (0.8)	0.4 (0.4)	0.8 (0.8)	0.1 (0.1)	NA (NA)	0.08 (0.08)
*Salmonella typhimurium* ATCC14028	0.5 (0.5)	NA (NA)	NA (NA)	0.8 (0.8)	NA (NA)	0.1 (0.1)	0.004 (0.008)	0.002 (0.002)
*Klebsiella pneumoniae* CAU202084	0.5 (0.5)	NA (NA)	NA (NA)	NA (NA)	NA (NA)	0.2 (0.2)	NA (NA)	0.001 (0.004)
*Proteus vulgaris* CVCC1971	NA (NA)	NA (NA)	NA (NA)	NA (NA)	NA (NA)	NA (NA)	NA (NA)	0.001 (0.004)
*Staphylococcus aureus* ATCC29213	0.5 (0.5)	NA (NA)	NA (NA)	0.4 (0.4)	0.8 (0.8)	0.1 (0.1)	0.002 (0.004)	0.004 (0.004)
*Staphylococcus haemolyticus* CAU202078	1 (1)	NA (NA)	NA (NA)	0.4 (0.4)	NA (NA)	0.1 (0.1)	NA (NA)	0.004 (0.004)
*Bacillus cereus* CAU 202020	1 (1)	NA (NA)	0.4 (0.4)	0.2 (0.2)	0.1 (0.1)	0.2 (0.2)	NA (NA)	0.004 (0.032)
*MRSA*^b^ ATCC33591	NA (NA)	NA (NA)	NA (NA)	NA (NA)	NA (NA)	NA (NA)	NA (NA)	0.004 (0.016)
*Streptococcus suis* CVCC3307	NA (NA)	NA (NA)	NA (NA)	NA (NA)	NA (NA)	NA (NA)	0.001 (0.002)	0.004 (0.008)

^a^ MIC is the minimum concentration which inhibits bacterial growth, MBC is the minimum concentration which kills 99.9% bacteria; ^b^ Methicillin- resistant *S. aureus*; ^c^ No antimicrobial activity at 1 mg/mL.

**Table 3:** Effects of temperature, extreme pH, serum, and protease on antimicrobial activity of Z-d14CFR (MIC, mg/mL).

	*E. coli* ATCC25922	*S. aureus* ATCC29213
37 °C	0.1	0.1
60 °C	0.1	0.1
80 °C	0.1	0.1
100 °C	0.1	0.1
121 °C	0.2	0.2
pH = 2	0.2	0.2
pH = 3	0.2	0.2
pH = 4	0.1	0.1
pH = 5	0.1	0.1
pH = 6	0.1	0.1
pH = 7	0.1	0.1
pH = 8	0.1	0.1
pH = 9	0.2	0.2
pH = 10	0.2	0.2
pH = 11	0.2	0.2
pH = 12	>2	0.2
10% FBS ^a^	0.2	0.2
UV exposure	0.1	0.1
Pepsin	>2	>2
Trypsin	>2	>2
Papain	>2	>2
α-chymotrypsin	>2	>2
proteinase K	>2	>2

^a^ Fetal bovine serum.

## Data Availability

Not applicable.

## Supplementary Materials

The following supporting information can be downloaded at: https://www.mdpi.com/article/10.3390/ijms23094617/s1.
