# Supplementary material for: A Novel β-Hairpin Peptide Z-d14CFR Enhances Multidrug-Resistant Bacterial Clearance in a Murine Model of Mastitis
Source: Int J Mol Sci. 2022 Apr 21;23(9):4617. doi: 10.3390/ijms23094617 (PMC9105976; doi:10.3390/ijms23094617)
Supplement: Supplementary file 1 [file ijms-23-04617-s001.zip › ijms-1669071-supplementary.pdf]

A Novel β-Hairpin Peptide Z-d14CFR Enhances Multidrug-Resistant Bacterial Clearance in a Murine Model of Mastitis

Table S1 Information of bacterial strains used in this study

Bacteria	Genotype	Source
Escherichia coli ATCC[1]25922		Purchased from China Institute of Veterinary Drug Center
Escherichia coli CVCC[2]1450		Purchased from China Institute of Veterinary Drug Center
Escherichia coli CAU 201919	(*tet(A)*, *bla*CTX-M-65, *aac*(3)-IId, *aph*(3")-Ib, *aph(3')-IIa*, *aph(6)-Id*, *formA*, *mdf(A)*, *sul*2)	Isolated from unpasteurized milk
Escherichia coli CAU 201920	(*tet(A)*, *aph*(3")-Ib, *aph(6)-Id*, *mdf*(A), *bla*TEM-1, *sul*2)	Isolated from unpasteurized milk
Salmonella typhimurium ATCC14028		Purchased from China Institute of Veterinary Drug Center
Klebsiella pneumoniae CAU202084	(*tet(A)*, *oqxAB*, *bla*CTX-M-3, *fosA5*)	Isolated from unpasteurized milk
Proteus vulgaris CVCC1971		Purchased from China Institute of Veterinary Drug Center
Staphylococcus aureus ATCC29213		Purchased from China Institute of Veterinary Drug Center
Staphylococcus haemolyticus CAU202078	(*tet(M)*, *drf (A)*, *ermC*, *blaZ*)	Isolated from unpasteurized milk
Bacillus cereus CAU 202020	(*tet(M)*, *bla*TEM-1, *sul*2)	Isolated from unpasteurized milk
MRSA[3] ATCC33591		Purchased from China Institute of Veterinary Drug Center
Streptococcus suis CVCC3307		Purchased from China Institute of Veterinary Drug Center

[1] American Type Culture Collection; [2] China Veterinary Culture Collection Center. [3] Methicillin-resistant *S. aureus*.

Table S2 Primers used for Real-Time PCR

Gene	Accession NO.	Prime sequence (5'-3')
β-actin	NM_007393.5	f- GCTCTTTTCCAGCCTTCCTT

		r- GATGTCAACGTCACACTT
IL1β	NM_008361.4	f- TGCCACCTTTTGACAGTGATG<br>r- AAGGTCCACGGGAAAGACAC
TNF-α	XM_006504297.5	f- ACGGCATGGATCTCAAAGAC<br>r-AGATAGCAAATCGGCTGACG

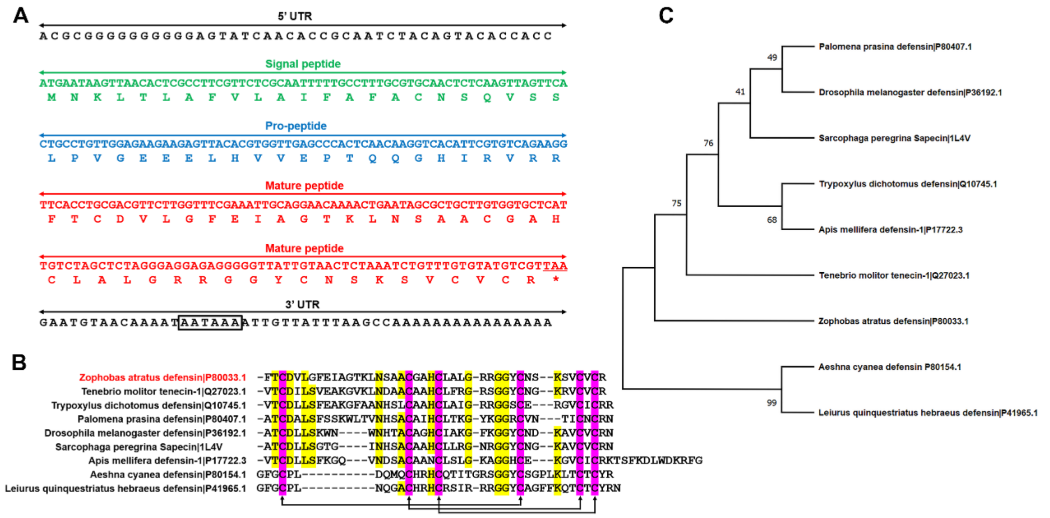

Figure S1 Amplification and analysis of *Z. atratus defensin*. (A) Analysis of *Z. atratus* defensin transcript, the 5' UTR and 3' UTR are marked with black, the putative polyA additional signal at 3' UTR (AATAAA) is boxed. The signal peptide, pro-peptide and mature peptide of *Z. atratus* defensin and their corresponding nucleotide sequence are respectively marked with green, blue and red. (B) Multiple sequence alignment of *Zophobas atratus* (*Z. atratus*) defensin. *Z. atratus* defensin (ZA-defensin) has six conserved cysteine residues like other insect defensins, and forms three disulfide bonds between Cys1-Cys4, Cys2-Cys5, Cys3-Cys6 (cysteines are shadowed in yellow and other identical residues are shadowed in grey). (C) A neighbor-joining distance tree

constructs from the alignment of defensin sequences presented in (B), numbers on interior branches represent bootstrap values

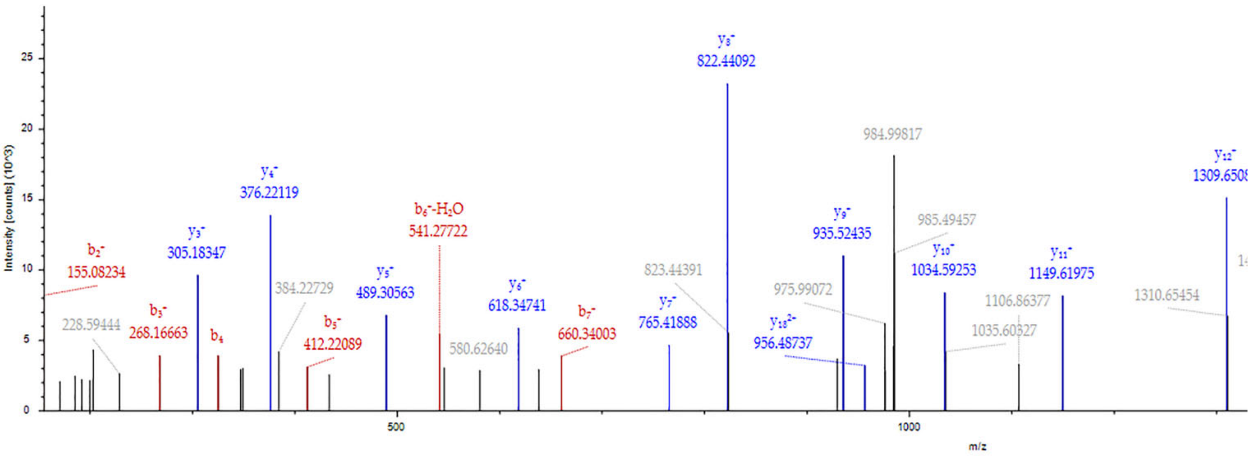

Figure S2 Mass spectrometry of recombinant *Z. atratus* defensin.

Z-d13

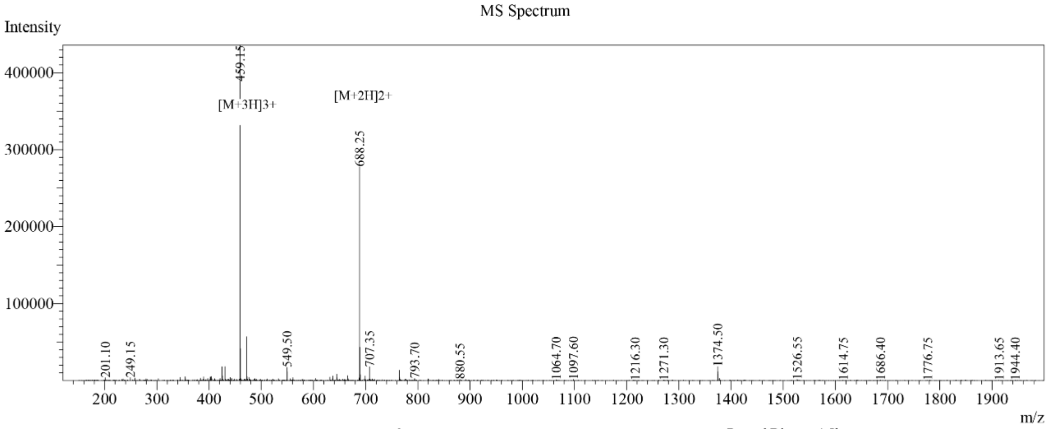

Z-d114C

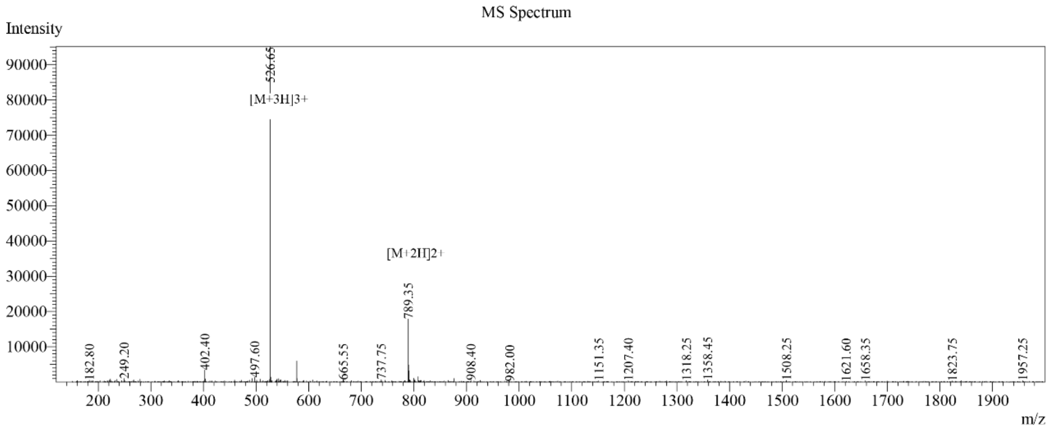

Z-d114CF

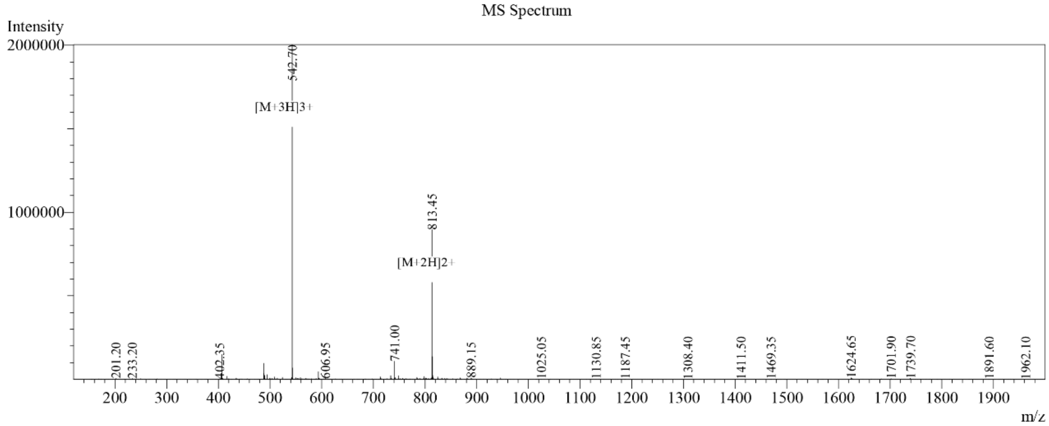

Z-d114CR

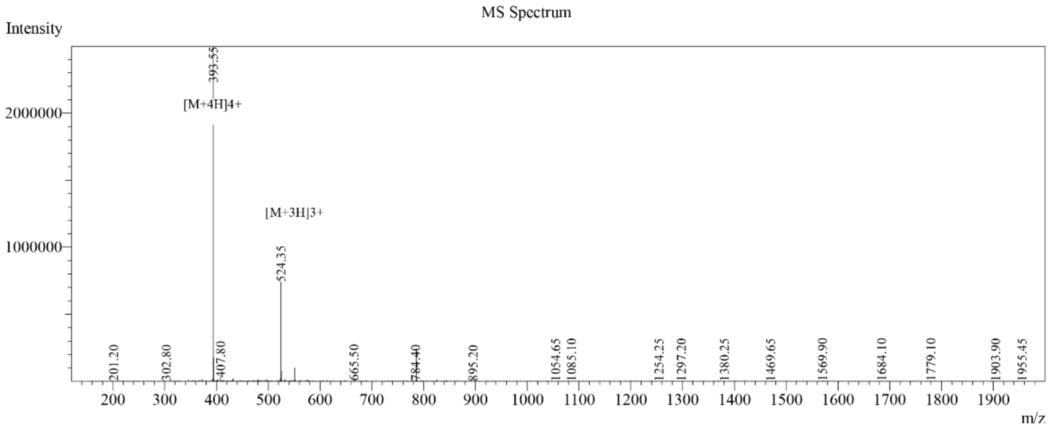

Z-d114CFR

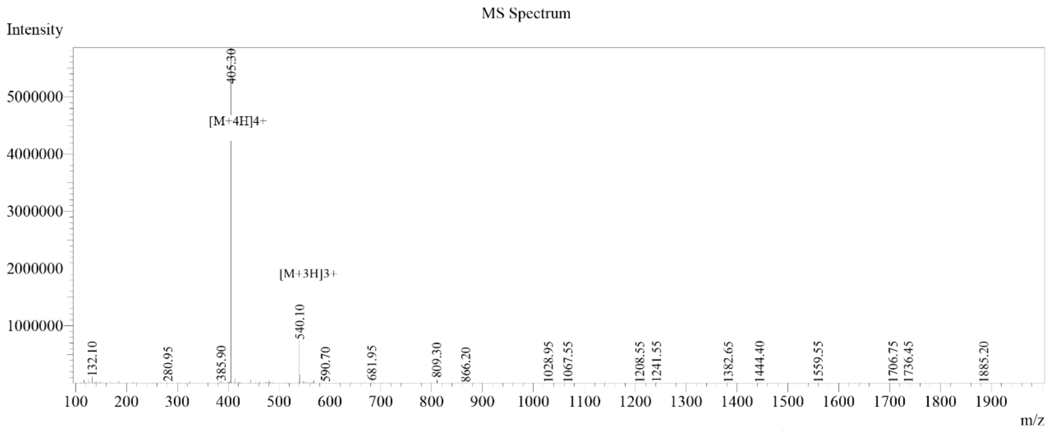

Figure S3 Mass spectrometry of the synthesized peptides.
